# Activation of a plasmid-situated type III PKS gene cluster by deletion of a *wbl* gene in deepsea-derived *Streptomyces somaliensis* SCSIO ZH66

**DOI:** 10.1186/s12934-016-0515-6

**Published:** 2016-06-27

**Authors:** Huiming Huang, Lukuan Hou, Huayue Li, Yanhong Qiu, Jianhua Ju, Wenli Li

**Affiliations:** Key Laboratory of Marine Drugs, Ministry of Education of China, School of Medicine and Pharmacy, Ocean University of China, Qingdao, 266003 China; CAS Key Laboratory of Marine Bio-resources Sustainable Utilization, Guangdong Key Laboratory of Marine Materia Medica, RNAM Center for Marine Microbiology, South China Sea Institute of Oceanology, Chinese Academy of Sciences, 164 West Xingang Road, Guangzhou, 510301 China

**Keywords:** Deepsea-derived *Streptomyces*, Cryptic gene cluster, *whiB*-like (*wbl*) gene, Violapyrones (VLPs), Type III polyketide synthase (PKS)

## Abstract

**Background:**

Actinomycete genome sequencing has disclosed a large number of cryptic secondary metabolite biosynthetic gene clusters. However, their unavailable or limited expression severely hampered the discovery of bioactive compounds. The *whiB*-like (*wbl*) regulatory genes play important roles in morphological differentiation as well as secondary metabolism; and hence the *wblA*_*so*_ gene was probed and set as the target to activate cryptic gene clusters in deepsea-derived *Streptomyces somaliensis* SCSIO ZH66.

**Results:**

*wblA*_*so*_ from deepsea-derived *S. somaliensis* SCSIO ZH66 was inactivated, leading to significant changes of secondary metabolites production in the *ΔwblA*_*so*_ mutant, from which α-pyrone compound violapyrone B (VLP B) was isolated. Subsequently, the VLP biosynthetic gene cluster was identified and characterized, which consists of a type III polyketide synthase (PKS) gene *vioA* and a regulatory gene *vioB*; delightedly, inactivation of *vioB* led to isolation of another four VLPs analogues, among which one was new and two exhibited improved anti-MRSA (methicillin-resistant *Staphylococcus aureus*, MRSA) activity than VLP B. Moreover, transcriptional analysis revealed that the expression levels of *whi* genes (*whiD*, *whiG*, *whiH* and *whiI*) and *wbl* genes (*wblC*, *wblE*, *wblH*, *wblI* and *wblK*) were repressed by different degrees, suggesting an intertwined regulation mechanism of *wblA*_*so*_ in morphological differentiation and secondary metabolism of *S. somaliensis* SCSIO ZH66.

**Conclusions:**

*wblA* orthologues would be effective targets for activation of cryptic gene clusters in marine-derived *Streptomyces* strains, notwithstanding the regulation mechanisms might be varied in different strains. Moreover, the availability of the *vio* gene cluster has enriched the diversity of type III PKSs, providing new opportunities to expand the chemical space of polyketides through biosynthetic engineering.

**Electronic supplementary material:**

The online version of this article (doi:10.1186/s12934-016-0515-6) contains supplementary material, which is available to authorized users.

## Background

Given marine environmental conditions are extremely different from the terrestrial environment, marine actinomycete strains have become an important source of pharmacologically active compounds [1]. Recently, microbial genome sequencing has brought to light a large number of cryptic secondary metabolite biosynthetic gene clusters, demonstrating the tremendous genetic potentials for producing secondary metabolites, which fundamentally refreshed the way for natural product discovery [2, 3]. However, most of these biosynthetic pathways are not expressed or only expressed in a very low titer under ordinary laboratory conditions, which severely hampered the discovery of bioactive compounds. Thus activation of cryptic gene clusters has become a tempting and rapidly developing field [4–6].

The control of antibiotic biosynthesis involves complex regulatory cascades and intertwined networks, and many of them are coordinately regulated together with the morphological differentiation [5, 7]. In general, regulators are classified as pathway-specific regulator (or cluster-situated regulator) and global regulator (or pleiotropic regulator) per their modes of action [5]. Among them, the *whiB*-like (*wbl*) regulatory genes have received much attention due to their diverse biological roles, such as in morphological differentiation and secondary metabolism [8, 9]. Wbls are small cytoplasmic proteins confined to actinobacteria, which contain four conserved cysteine residues coordinating an Fe-S cluster [8, 10]. The chromosome of *Streptomyces coelicolor* A3(2) contains 11 *wbl* genes: *whiB* and *whiD* genes are involved in sporulation; *wblA* controls major development transitions; *wblC* mutant was hypersensitive to a wide range of antibiotics; inactivation of *wblE* was lethal; no obvious effects were observed when the other six *wbl* genes (*wblH*, -*I*, -*J,* -*K*, -*L*, -*M*) were inactivated [8]. Notably, *wblA* and its homologues have been reported to serve as global regulators for the biosynthesis of various antibiotics, mostly in a negative manner (such as for actinorhodin [8], tautomycetin [11] and doxorubicin [12]); conversely, it was found to exert dual function in antibiotic biosynthesis in *Streptomyces chattanoogensis* L10 [13] and *Streptomyces ansochromogenes* 7100 [14] as well.

**Fig. 1:**
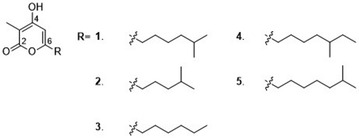
The chemical structures of violapyrones (VLPs 1-5). **1**, VLP B; **2**, VLP A; **3**, VLP J; **4**, VLP C; **5**, VLP H. **3** is a novel VLP analogue isolated from the *ΔvioB* mutant

In our efforts to discover novel natural products from marine *Streptomyces* strains by using genome mining strategy, *wblA*_*so*_ gene was set as the target to activate cryptic gene clusters in the deepsea-derived *Streptomyces somaliensis* SCSIO ZH66, leading to significant changes of secondary metabolites production in the *ΔwblA*_*so*_ mutant, from which α-pyrone compound violapyrone B (VLP B, **1**, Fig. 1) was isolated and identified. VLPs were first isolated from *Streptomyces violascens* YIM 100525 obtained from *Hylobates hoolock* feces, and were reported to inhibit the growth of *Bacillus subtilis* and *Staphylococcus aureus* [15]. Simultaneously, VLPs were also named as presulficidins, which serve as sulfate donors relaying sulfonate from 3′-phosphoadenosine 5′-phosphosulfate (PAPS) to caprazamycin [16].

Pyrones are usually assembled by type III polyketide synthases (PKSs) [16–20], which are homodimeric ketosynthases that catalyze condensation of one to several molecules of extender substrate onto a starter substrate through iterative decarboxylative Claisen condensation reactions [21]. Notably, type III PKSs usually exhibit broad substrate promiscuity, and can recognize unnatural substrates to generate novel unnatural products, which renders them excellent candidates for enzymatic engineering to expand chemical space of polyketides [22, 23]. Several pyrone-encoding type III PKS have been identified. For instance, BpsA synthesizes of triketide pyrones from long-chain fatty acyl-CoA thioesters as starter substrates and malonyl-CoA as extender substrate in *B. subtilis* [17]; Gcs catalyzes the condensation of an acyl carrier protein (ACP) ester of β-keto acid and ethylmalonyl-CoA to form germicidins in *S. coelicolor* A3(2) [18, 23]; DpyA catalyzes the synthesis of alkyldihydropyrones using β-hydroxyl acid thioesters as starter substrates in *Streptomyces reveromyceticus* [19]; ArsC accepted several acyl-CoAs with various lengths of the side chain as a starter substrate to give corresponding alkylpyrones [20]; Cpz6 was demonstrated to encode presulficidins in vivo putatively from CoA- or ACP-activated iso-acyl starter units as well as one malonyl and one methylmalonyl unit in *Streptomyces* sp. MK730–62F2 [16].

Herein, VLP B (**1**) was found via inactivation of the global regulatory gene *wblA*_*so*_ from deepsea-derived *S. somaliensis* SCSIO ZH66; the type III PKS gene *vioA* encoding VLP was then identified; subsequently, the VLP biosynthetic gene cluster was characterized, leading to isolation of another four VLPs compounds (**2**–**5**), among which one was new (**3**) and notably two (**4** and **5**) exhibited improved anti-MRSA (methicillin-resistant *S. aureus*, MRSA) activity than **1**.

## Results

### Inactivation of *wblA*_*so*_ led to significant enhancement of VLP B production

**Fig. 2:**
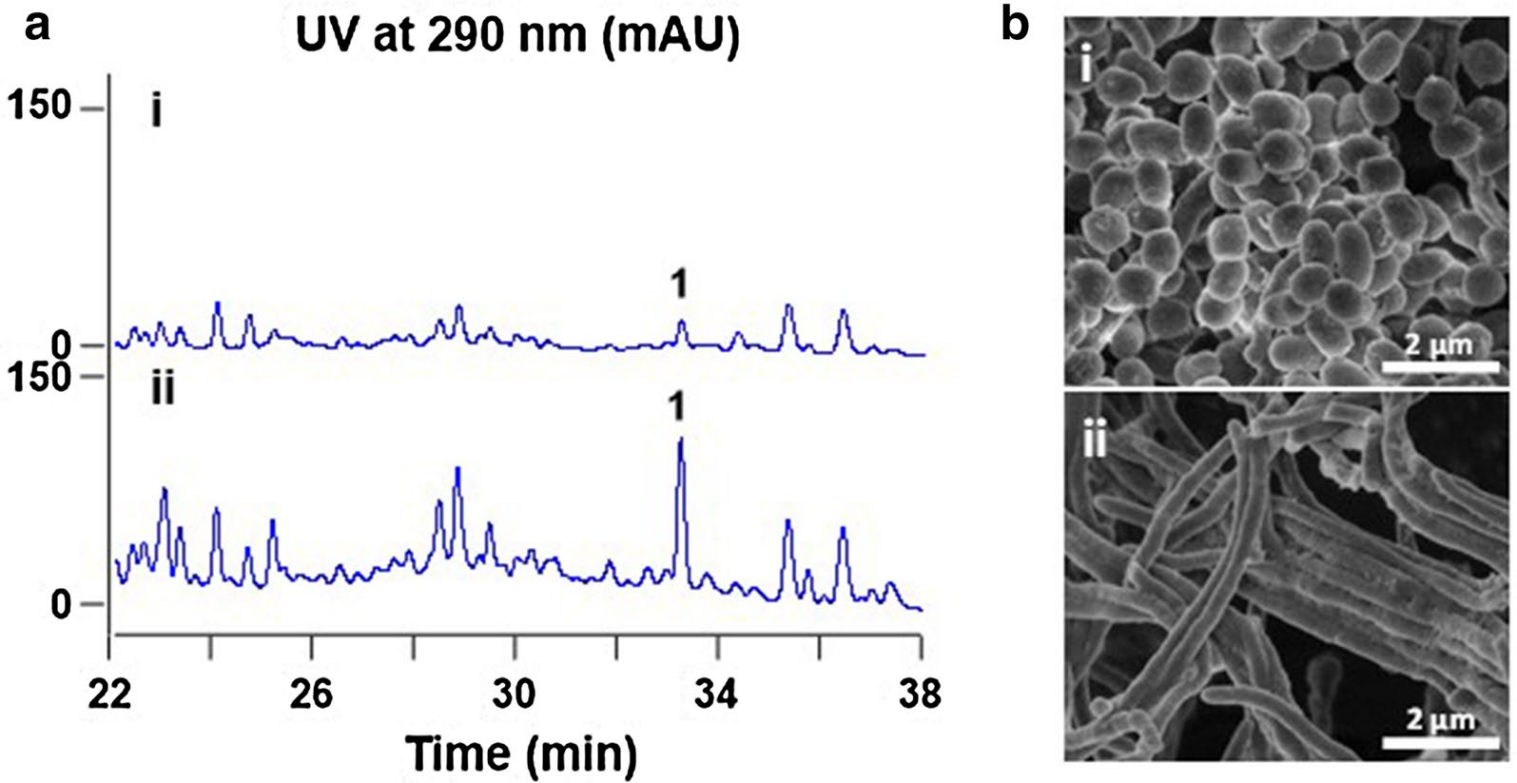
Comparisons of the wild-type strain and the mutant strain *ΔwblA*_*so*_. **a** HPLC traces of the fermentation broths from the wild-type strain (i) and the mutant strain *ΔwblA*_*so*_ (ii). “**1**″ indicates the accumulated compound VLP B by *ΔwblA*_*so*_. **b** The scanning electron micrographs of the wild-type strain (i) and the mutant strain *ΔwblA*_*so*_ (ii) after incubation on MS plate at 30 °C for 4 days

As *wblA* and its orthologous genes were reported to be down-regulators for secondary metabolism in *Streptomyces* strains [11, 12, 24, 25], the orthologous gene designated *wblA*_*so*_ was probed from *S. somaliensis* SCSIO ZH66 and was set as a target for activating cryptic gene clusters. WblA_so_ exhibited 85 % identity to WblA (CAB43030.1) from *S. coelicolor* A3(2) with a highly conserved Cys-X_21_-Cys-X_2_-Cys-X_5_-Cys motif, which coordinate a redox–sensitive iron-sulphur cluster [10]. Gene inactivation was performed to detect its impact on production of secondary metabolites in *S. somaliensis* SCSIO ZH66. The *ΔwblA*_*so*_ mutant was obtained as described in the “Methods” section. After confirmation by PCR analysis (Additional file 1: Figure S1), fermentations were carried out and the accumulated metabolites were analyzed by high-pressure liquid chromatography (HPLC). As shown in Fig. 2a, the profile of the *ΔwblA*_*so*_ mutant (panel ii) substantially changed and productions were enhanced compared to those in the wild-type strain (panel i), indicating that WblA_so_ functions as a global negative regulator for secondary metabolism in *S. somaliensis* SCSIO ZH66. Compound **1** at retention time of 33.1 min, which was a significantly enhanced peak (by about fivefold) at wavelength of 290 nm, was then purified and subject to structural analysis. The UV spectrum of **1** displayed λ_max_ at 290 nm (Additional file 1: Figure S2A), and the chemical formula of **1** was determined to be C_13_H_2_O_3_ by high-resolution electrospray mass spectrometry (HR-ESI–MS) (*m/z* 225.1483 [M + H]^+^, calcd 225.1491) (Additional file 1: Figure S2B). The ^1^H NMR data of **1** was further recorded (Additional file 1: Figure S2C), leading to its identification as antibiotic VLP B (Fig. 1) by comparison of all the above data with those previously reported [15]. VLP B was also named as presulficidin A, which relays sulfonate from PAPS to caprazamycin [16].

### Identification of the type III PKS gene involved in VLP B biosynthesis

**Fig. 3:**
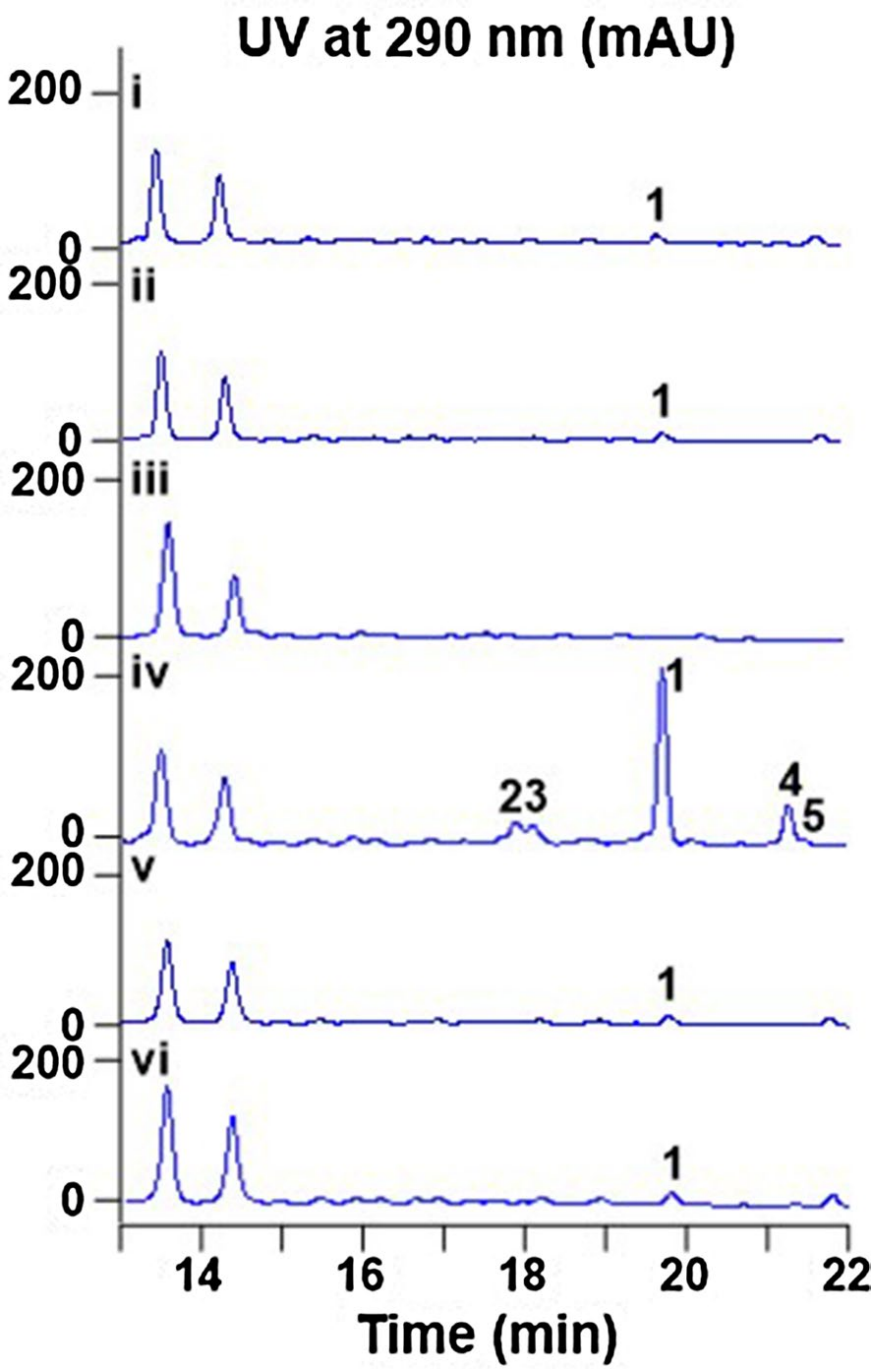
HPLC traces of the fermentation broths from *S. somaliensis* SCSIO ZH66 strains. (i) the wild-type strain; (ii) *ΔpksIII*-*1*; (iii) *ΔvioA*; (iv) *ΔvioB*; (v) *Δorf1*; (vi) *Δorf(*-*1*-*2)*

To identify the VLP biosynthetic gene cluster, bioinformatic analysis of the *S. somaliensis* SCSIO ZH66 genome was performed, revealing the presence of two type III PKS genes, *pksIII*-*1* and *pksIII*-*2*. BlastP searches against the GenBank nr database revealed that PksIII-1 exhibited 72 % identity to the 1,3,6,8-tetrahydroxynaphthaene synthase (THNS) from *S. coelicolor* A3(2) (WP_011027653.1), and PksIII-2 displayed highly homology (95 % identity) to a putative THNS from *Streptomyces* sp. W9 (WP_012840496.1, Table 1). Further sequence alignment of PksIII-2 with reported pyrone synthases indicated that PksIII-2 exhibited 53 % identity to Cpz6 and 36 % identity to DpyA (BAQ19510.1), suggesting PksIII-2 might encode VLP B in *S. somaliensis* SCSIO ZH66. Both *pksIII*-*1* and *pksIII*-*2* were inactivated, resulting in *ΔpksIII*-*1* and *ΔpksIII*-*2* mutants (Additional file 1: Figure S3 and S4). HPLC analysis of their fermentation products showed that *ΔpksIII*-*2* failed to accumulate **1** (Fig. 3 panel iii), while the *ΔpksIII*-*1* mutant (Fig. 3 panel ii) produced the same little amount of **1** as that of the wild type strain (Fig. 3 panel i). This result demonstrated that it is *pksIII*-*2* that is involved in VLP B biosynthesis, and thus, *pksIII*-*2* was renamed as *vioA*.

**Table 1:** Proposed functions of proteins encoded by the *vio* biosynthetic gene cluster in *S. somaliensis* SCSIO ZH66

Protein	Size (aa)	Proposed function	Homologs	
			Protein/organism	Accession no. (identity/similarity %)
Orf(-3)	88	Hypothetical protein	pCQ3_109/*Streptomyces* sp. W9	WP_012840495.1 (65/70)
Orf(-2)	63	Hypothetical protein	SVEN_2103/*Streptomyces venezuelae* ATCC 10712	WP_015033307.1 (60/66)
Orf(-1)	70	Hypothetical protein	pCQ4.14/*Streptomyces* sp. W75	WP_015060953.1 (50/52)
VioA	350	Type III PKS	pCQ3.110c/*Streptomyces* sp. W9	WP_012840496.1 (95/97)
VioB	272	XRE-family regulator	pCQ4.19c/*Streptomyces* sp. W75	WP_015060958.1 (95/95)
Orf1	158	Hypothetical protein	pCQ3.4c/*Streptomyces* sp. W9	WP_012840390.1 (61/62)
Orf2	76	Hypothetical protein	pCQ4.20c/*Streptomyces* sp. W75	WP_015060959.1 (31/34)

### Characterization of the *vio* gene cluster

**Fig. 4:**
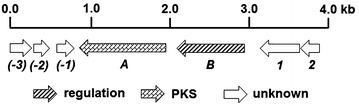
Genetic organization of the *vio* gene cluster. Proposed functions of individual open reading frames are coded with various patterns and summarized in Table 1

We then analyzed the surrounding sequences of *vioA*, and found that this gene is situated in a circular plasmid with the size of ~85 kb. Adjacent to *vioA*, a regulatory gene *vioB* was found, which displayed 94 % identity to an unknown regulator from *Streptomyces* sp. W9 (WP_012840387.1); Orf1-Orf2 and Orf(-1)-Orf(-3) are hypothetical proteins with unknown functions (Fig. 4, Table 1). Gene inactivation was performed to investigate their functions (Additional file 1: Figure S5–S7). As shown in Fig. 3, inactivation of *vioB* led to production enhancement of **1** by about eightfold (panel iv), suggesting it serves as a negative regulator; while inactivation of *orf1* (panel v) and *orf(*-*1*-*2)* (panel vi) had no obvious impacts on **1** production, indicating that they are probably beyond the gene cluster. Therefore, the *vio* gene cluster consists of only two genes, the structural gene *vioA* and the regulatory gene *vioB*.

### Identification of VLP analogues with improved anti-MRSA activity

**Fig. 5:**
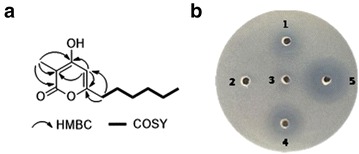
Correlations of compound **3** and bioassays of VLPs. **a** Key HMBC and COSY correlations of **3** in DMSO-*d6*. **b** Antibacterial activity of VLPs against MRSA. 100 µg of each compound dissolved in methanol was added. Inhibition zones were observed after incubation at 37 °C for 20 h; methanol has no impact on the growth of MRSA

Careful analysis of the *ΔvioB* mutant revealed that a few more VLP analogues were accumulated as well in addition to **1** (Fig. 3, panel iv). Therefore, large scale fermentations were performed, leading to isolation of another 4 VLPs (**2**–**5**) (Fig. 1). The molecular formula of **3** was C_12_H_18_O_3_, as determined by HR-ESI–MS (*m/z* 211.1328 [M + H]^+^, calcd 211.1334) (Additional file 1: Figure S8A), having less CH_2_ unit than that of **1**. Full sets of 1D and 2D NMR spectra of **3** were acquired, thereby allowing us to complete its structure assignments (Table 2; Fig. 5a, Additional file 1: Figure S8). According to the COSY and HMBC correlations, **3** has the same 3-methyl-4-hydroxy-α-pyrone backbone as that in **1**, and the side chain at C-6 was assigned as hexyl (Fig. 5a). Thus, **3** was identified to be a novel VLP analogue, named VLP J (Fig. 1). Compounds **2**, **4** and **5** were identified as VLP A, VLP C and VLP H, respectively, by comparison of their HR-ESI–MS and ^1^H NMR data with those of reported (Additional file 1: Figure S9–S11) [15, 26]. Inspection of the structure of VLPs **1**–**5** suggests that they are probably assembled from different CoA- or ACP-tethered β-keto acids from branched-chain (for **1**, **2**, **4** and **5**) or straight-chain (for **3**) fatty acid metabolism and methylmalonyl CoA via Claisen condensation.

**Table 2:** Assignments from 500 MHz NMR spectroscopies of **3** in DMSO-*d*_*6*_ 
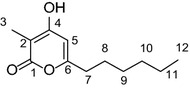

Position	Compound 3	
	δ_H_ (*J* in Hz)	δ_C_
1	–	165.2
2	–	95.5
3	1.71 (s)	8.3
4	–	166.7
5	5.91 (bs)	100.2
6	–	161.9
7	2.37 (t, 7.5)	32.6
8	1.51 (m)	26.1
9	1.27 (m)	27.6
10	1.26 (m)	30.9
11	1.26 (m)	21.7
12	0.85 (t, 6.5)	13.7

Furthermore, the anti-MRSA bioactivities of VLPs **1**–**5** were investigated, and the result revealed that all of them with the exception of **2** showed inhibition against MRSA at the concentration of 100 μg/well; compound **5** with a minimum inhibitory concentration (MIC) value of 25 μg/mL gave the best activity among the VLPs tested (Fig. 5b, Additional file 1: Table S1). These results demonstrated that the polarity of the VLPs, which are mostly up to the length of alkyl side chains, plays an essential role for their anti-MRSA activity.

### Effects of *wblA*_*so*_ gene inactivation on the *vio* gene cluster

**Fig. 6:**
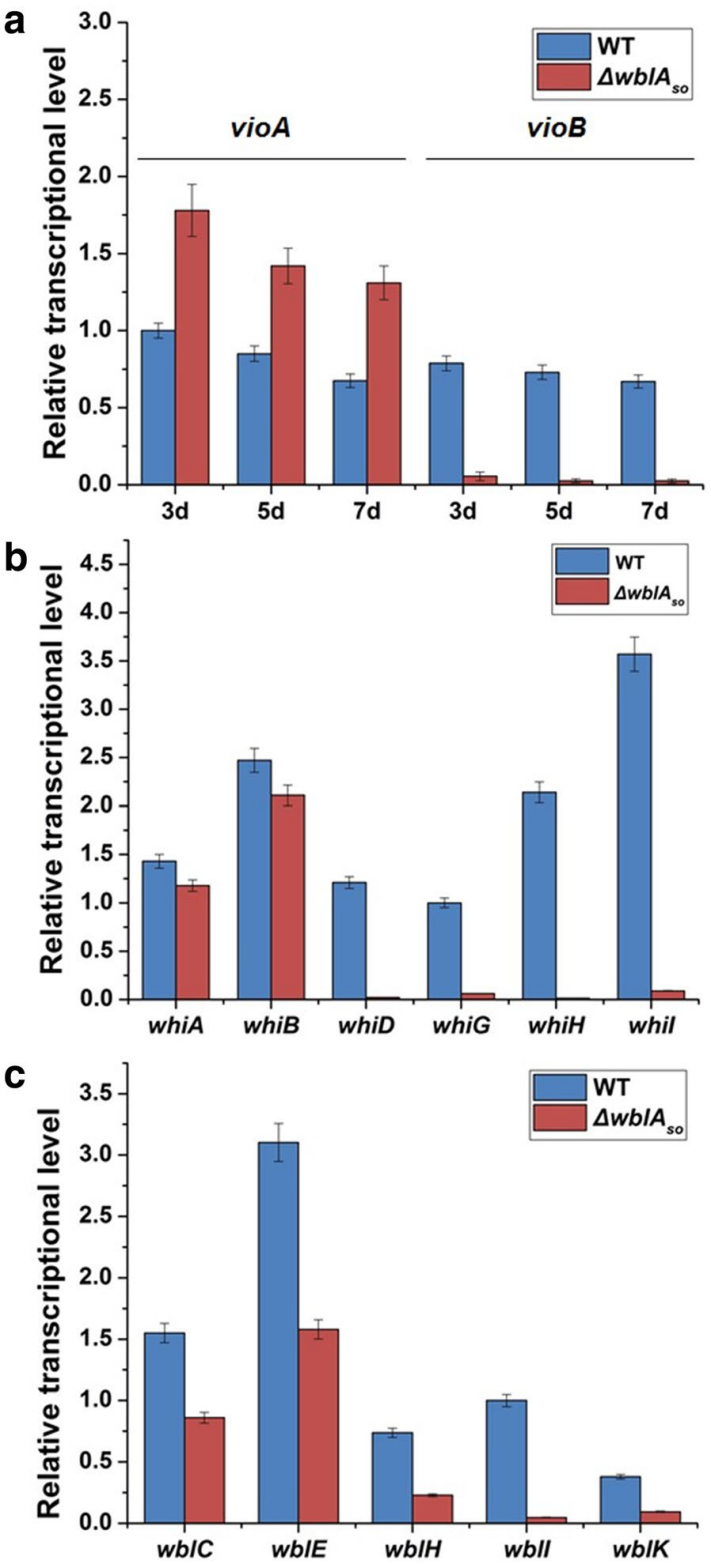
Effects of *wblA*_*so*_ inactivation on the expression levels of the *vio* (**a**), *whi* (**b**) and *wbl* (**c**) genes. For the *vio* genes, the transcription levels were detected at 3, 5 and 7 days in the wild-type strain and the mutant strain *ΔwblA*_*so*_ cultured in fermentation medium at 30 °C. For the *whi* and *wbl* genes, the transcription levels were detected in the wild-type strain and the *ΔwblA*_*so*_ mutant grown on MS plates at 30 °C for 4 days. The transcription level of *hrdB* was used as an internal control. Error bars indicated standard deviations (n = 3)

To detect the effects of *wblA*_*so*_ on the expression of the *vio* gene cluster, the transcription levels of *vioA* and *vioB* were analyzed by quantitative real-time RT-PCR (qPCR). As shown in Fig. 6a, transcription of *vioB* was substantially reduced in the *ΔwblA*_so_ mutant in comparison to the wild-type strain during fermentation; simultaneously, expression of *vioA* was obviously enhanced, suggesting that activation of the *vio* gene cluster was probably achieved via repression of *vioB*. We next set out to determine whether WblA_so_ regulated the *vio* gene cluster directly. To this end, we performed electrophoretic mobility shift assay (EMSA) to detect the binding ability of WblA_so_ to the promoter regions of *vioA* and *vioB* under anaerobic conditions; however, no binding was observed (data not shown).

### Effects of *wblA*_*so*_ inactivation on morphology of *S. somaliensis* SCSIO ZH66

Moreover, the effects of *wblA*_*so*_ inactivation on morphological development were investigated, and the phenotype of the *ΔwblA*_*so*_ mutant was compared to the wild-type strain on mannitol-soy flour (MS) medium. While the wild-type strain sporulated well when incubated on MS plate at 30 °C for 4 days, the *ΔwblA*_*so*_ mutant defected in sporulation at the same conditions (Additional file 1: Figure S12). Scanning electron microscopy of the surfaces of the strains revealed abundant spore chains of the wild-type strain (Fig. 2b, panel i), in contrast, thin and sparse aerial hyphae of the *ΔwblA*_*so*_ mutant (Fig. 2b, panel ii), consistent with previous observations in other *Streptomyces* strains [8, 13, 14]. We further evaluated the transcription levels of *whi* genes as well as other *wbl* genes. As shown in Fig. 6b, the transcription of *whiD* and *whiH* was almost abolished in the *ΔwblA*_*so*_ mutant; the transcription levels of *whiG* and *whiI* were severely decreased to ~6 % of the wild-type levels; conversely, only slight difference were observed for the transcription levels of *whiA* (~85 % of the wild-type level) and *whiB* (~82 % of the wild-type level). Interestingly, the transcription levels of the other *wbl* genes (*wblC*, *wblE*, *wblH*, *wblI* and *wblK*) were also significantly decreased to ~5–55 % of the wild-type levels (Fig. 6c). These findings implied that *wblA*_*so*_ served as a multifunctional regulator via a very complex network involved in *whi* genes as well as other *wbl* genes.

## Discussion

Manipulation of global regulators was one of the effective strategies for activation of cryptic secondary metabolite biosynthetic gene clusters [5, 6]. However, the orthologues of a specific regulator can play distinct roles in different biological backgrounds. Marine microorganisms have been endowed with unique physiological functions, and thereby unusual metabolic pathways during evolution in specific ecological environments [1], indicating their underlying regulation mechanisms might be unique as well. In the present study, the global regulatory gene *wblA*_*so*_ was discerned from deepsea-derived *S. somaliensis* SCSIO ZH66, and was then deleted to activate cryptic gene clusters, leading to identification of anti-MRSA compound VLP B and thereafter its encoding gene cluster.

So far, the regulatory mechanisms of WblA and its orthologues in antibiotics biosynthesis are still unknown [13, 14]. WblAs all harbor a conserved helix-turn-helix DNA-binding motif, indicating they probably function by binding the promotor regions of the target genes. However, no evidences have been obtained to support their binding abilities to antibiotic biosynthetic genes [13]. Further efforts need to be devoted to clarify their mechanisms executing regulation of antibiotics biosynthesis. In *Streptomyces* species, production of secondary metabolites is closely coordinated with morphological differentiation [5, 7]. Inactivation of *wblA* and its orthologous genes had effects on both processes [8, 13, 14], and hence the transcription levels of *whi* genes as well as other *wbl* genes were investigated in this study (Fig. 6). Interestingly, transcription evaluation of the *whi* genes in the *ΔwblA*_*ch*_ mutant suggested *whiB*, *whiH* and *whiI* were nearly not transcripted; the expression of *whiA* was decreased to about ~20 % of the wild-type level; on the contrary, the transcription level of *whiD* was increased slightly [13]. However, our result revealed that transcription levels of not only *whiH* and *whiI* but also *whiD* and *whiG* were all severely decreased in the *ΔwblA*_*so*_ mutant compared to those in the wild-type strain; conversely, the expression of *whiA* and *whiB* was only decreased slightly (Fig. 6b). The different impacts caused by inactivation of *wblA* orthologues probably implied their varied regulation mechanisms in different strains. It is worth to mention that the transcription levels of other *wbl* genes in the *ΔwblA*_*so*_ mutant were also decreased by different degrees (Fig. 6c), suggesting *wblA*_*so*_ probably interacts with other *wbl* genes as well. Thus, we could speculate that *wblA*_*so*_ is a multifunctional regulator with an intertwined and sophisticated mechanism.

Although both Cpz6 and VioA catalyze the formation of presulficidin A/VLP B, the genetic contexts of *cpz6* and *vioA* are totally different: *cpz6* is situated in the caprazamycin biosynthetic gene cluster, and *vioA* lies in the *vio* gene cluster consisting of only two genes; in addition, VioA encodes VLPs with different types and ratios from presulficidins synthesized by Cpz6 [16]. These facts suggest VLPs might serve different biological functions in *S. somaliensis* SCSIO ZH66 from presulficidins in *Streptomyces* sp. MK730–62F2, which relay sulfonate from PAPS to caprazamycin [16]. The different types and ratios of the products encoded by Cpz6 and VioA might be dictated by their different substrate preference as well as substrate availability in different biological backgrounds.

Here, for the first time, VLPs were shown to display anti-MRSA activity (Fig. 5b). As compounds **1**–**5** all have the same 3-methyl-4-hydroxy-α-pyrone backbone, the differences in their anti-MRSA activity can be ascribed to the influence of the alkyl side chain at C-6 (Fig. 1). As shown in Fig. 5b, the anti-MRSA activity increased with decrease in the polarity of the compounds, suggesting that the lipophilic nature of the alkyl chain plays an important role for the activity. These findings pointed out prospective directions for bioactivity improvement of VLPs. Further exploration of substrate promiscuity of VioA towards unnatural malonyl-CoA analogues would provide more opportunities to engineer chemical diverse polyketides using rational approaches.

## Conclusions

A plasmid-situated type III PKS gene cluster was activated by deletion of the *wblA*_*so*_ gene in deepsea-derived *S. somaliensis* SCSIO ZH66, leading to isolation of anti-MRSA α-pyrone compound **1**. Further identification and characterization of the *vio* gene cluster resulted in one novel VLP analogue (**3**) and two VLPs analogues (**4** and **5**) with improved anti-MRSA bioactivity than that of **1**. Therefore, *wblA* orthologues would be effective targets for activation of cryptic gene clusters in marine-derived *Streptomyces* strains. In addition, the availability of the *vio* gene cluster has enriched the diversity of type III PKSs, providing additional opportunities to biosynthetically engineer chemical diverse polyketides for drug development.

## Methods

### Bacterial strains, plasmids, and culture conditions

All strains and plasmids used in this study are listed in Additional file 1: Table S2. *Escherichia coli* DH5α was served as the host for general subcloning [27]. *Escherichia coli* Top10 (Invitrogen, Carlsbad, La Jolla, CA, USA) was used as the transduction host for cosmid library construction. *Escherichia coli* ET12567/pUZ8002 [28] was used as the cosmid donor host for *E. coli*-*Streptomyces* intergenic conjugation. *Escherichia coli* BW25113/pIJ790 was used for λRED-mediated PCR-targeting [29]. The *S. somaliensis* SCSIO ZH66 (CGMCC NO. 9492) was isolated from the deep sea sediment collected at a depth of 3536 meters of the South China Sea (120° 0.250′E; 20° 22.971′N), and has been described previously [30]. *E. coli* strains were routinely cultured in Luria–Bertani (LB) liquid medium at 37 °C, 200 rpm, or LB agar plate at 37 °C. *Streptomyces* strains were grown at 30 °C on MS medium for sporulation and conjugation, and were cultured in tryptic soy broth (TSB) medium for genomic DNA preparation. Fermentation medium consists of 1 % soluble starch, 2 % glucose, 4 % corn syrup, 1 % yeast extract, 0.3 % beef extract, 0.05 % MgSO_4_·7H_2_O, 0.05 % KH_2_PO_4_, 0.2 % CaCO_3_, and 3 % sea salt, pH = 7.0, which was further supplemented with 1.5 % XAD-16 resin when fermenting the *ΔwblA*_*so*_ mutant.

### DNA isolation and manipulation

Plasmid extractions and DNA purifications were carried out using standardized commercial kits (OMEGA, Bio-Tek, Guangzhou, China). PCR reactions were carried out using Pfu DNA polymerase (TIANGEN, Beijing, China). Oligonucleotide synthesis and DNA sequencing were performed by Sunny Biotech company (Shanghai, China). Restriction endonucleases and T4 DNA ligase were purchased from Fermentas (Shenzhen, China).

### Genomic library construction and library screening

*S. somaliensis* SCSIO ZH66 genomic DNA was partially digested with *Sau*3AI, and fragments with the size of 40-50 kb were recovered and dephosphorylated with CIAP, and then ligated into SuperCos1 that was pretreated with *Xba*I, dephosphorylated, and digested with *Bam*HI. The ligation product was packaged into lambda particles with the MaxPlax Lambda Packaging Extract (Epicenter, Madison, WI, USA) as per the manufacture’s instruction and plated on *E. coli* Top10. The titer of the primary library was about 2 × 10^6^ cfu per μg of DNA. Specific primers were designed per the draft genome sequence for library screening against 2500 colonies by PCR (Additional file 1: Table S3).

### Sequence analysis

The two type III PKSs were identified from the *S. somaliensis* SCSIO ZH66 genome using the antiSMASH program [31]. *orf* assignments and their proposed function were accomplished by using the FramePlot 4.0beta (http://nocardia.nih.go.jp/fp4) [32] and Blast programs (http://blast.ncbi.nlm.nih.gov/Blast.cgi) [33], respectively.

### Gene inactivation

Gene inactivation in *S. somaliensis* SCSIO ZH66 was performed using the REDIRECT Technology according to the literature protocol [29, 34]. The amplified *aac(3)IV*-*oriT* resistance cassette from pIJ773 was transformed into *E. coli* BW25113/pIJ790 containing corresponding cosmid to replace an internal region of the target gene. Mutant cosmids were constructed (Additional file 1: Table S4) and introduced into *S. somaliensis* SCSIO ZH66 by conjugation from *E. coli* ET12567/pUZ8002 according to the reported procedure [35]. The desired mutants were selected by the apramycin-resistant and kanamycin-sensitive phenotype, and were further confirmed by PCR (Additional file 1: Table S5, Figures. S1, S3–S7).

### Production and analyses of VLPs

The fermentation cultures were harvested by centrifugation, and the supernatant was extracted twice with an equal volume of ethyl acetate. The combined EtOAc extracts were concentrated in vacuo to afford residue A. In the case of the *ΔwblA*_*so*_ mutant, the precipitated mycelia and XAD-16 resin were extracted twice with acetone. The extracts were combined, and acetone was evaporated in vacuo to yield residue B. The combined residues (for the *ΔwblA*_*so*_ mutant) or residue A (for the other mutants) were dissolved in MeOH, filtered through a 0.2 μm filter, and subject to HPLC. The HPLC system consisted of Agilent 1260 Infinity Quaternary pumps and a 1260 Infinity diode-array detector. Analytical HPLC was performed on an Eclipse C18 column (5 μm, 4.6 × 150 mm) developed with a linear gradient from 5 to 80 % B/A in 40 min (for analyzing *ΔwblA*_*so*_ mutant) or 20 to 70 % B/A in 20 min (for analyzing all the other mutants reported here) (phase A: 0.1 % formic acid in H_2_O; phase B: 100 % acetonitrile supplemented with 0.1 % formic acid) followed by an additional 10 min at 100 % B at flow rate of 1 mL/min and UV detection at 290 nm. For VLPs purification, semi-preparative HPLC was carried out using an YMC-Pack ODS-A C18 column (5 μm, 120 nm, 250 × 10 mm). Samples were eluted with a linear gradient from 50 to 80 % B/A in 40 min, followed by 100 % B for 10 min at a flow rate of 2.0 mL/min and UV detection at 290 nm. The identity of VLPs were confirmed by HR-ESI–MS and NMR analysis. HR-ESI-MS was carried out on Thermo LTQ-XL mass spectrometer. NMR data was recorded with an Agilent-DD2 500 spectrometer.

### Microscopy

For scanning electron microscopy, colonies were fixed in 2.5 % (v/v) glutaraldehyde at 4 °C overnight, stained with osmic acid for 2–4 h and dehydrated with ethanol at different concentrations. Each sample was coated with platinum-gold and then detected using a Hitachi S-4800 scanning microscope.

### Biological assays

The antibacterial activity of VLPs was assayed by agar diffusion test against methicillin-resistant *S. aureus* CCARM 3090. The MRSA strain was seeded in LB medium and then incubated at 37 °C for 20 h. After dilution with LB to 10^8^ cfu/mL, 25 μL of cell suspension was mixed with 25 mL LB medium for each plate. Subsequently, 10 μL of VLPs, at a final concentration of 10 mg/mL, were added to the sample wells and the inhibition zones were observed after incubation at 37 °C for 20 h. For determination of MIC values, the VLPs solutions were prepared in methanol and dispensed into 96-well plates using serial dilution method. Different concentration ranges were used for each compound. The overnight culture of MRSA was diluted to 10^6^ cfu/mL when used. LB broth was used as a blank control, and methanol and tetracycline were used as a negative control and a positive control, respectively. The growth of MRSA was measured after 12 h of incubation at 30 °C on a microplate reader (Epoch2, Biotech) at wavelength of 600 nm. Each assay was performed in triplicate.

### Transcriptional analysis by quantitative real-time RT-PCR

Total RNAs were prepared using Ultrapure RNA Kit (CWBio. Inc., Beijing, China). qPCR was performed as described previously [30]. The primers for qPCR are listed in Table S6.

### Nucleotide sequence accession number

The nucleotide sequences of *wblA*_*so*_, *pksIII*-*1* and the *vio* gene cluster reported in this paper have been deposited in the GenBank database under accession numbers of KU534996, KU534994 and KU534995, respectively.

## Additional file

10.1186/s12934-016-0515-6 Anti-MRSA activities of violapyrones (VLPs **1**–**5**). **Table S2.** Bacteria and plasmids used in this study. **Table S3.** The primer pairs used for cosmid library screening. **Table S4.** The primer pairs used for PCR-targeted mutagenesis. **Table S5.** The primer pairs used for PCR confirmation of the mutants. **Table S6.** The primer pairs used for qPCR analysis. **Figure S1.** Inactivation of *wblA*_*so*_. **Figure S2.** Spectral data of VLP B, **1**. **Figure S3**. Inactivation of *pksIII*-*1*. **Figure S4.** Inactivation of *vioA*. **Figure S5.** Inactivation of *vioB*. **Figure S6.** Inactivation of *orf1*. **Figure S7.** Inactivation of *orf(*-*1*-*2)*. **Figure S8.** Spectral data of VLP J, **3**. **Figure S9.** Spectral data of VLP A, **2**. **Figure S10.** Spectral data of VLP C, **4**. **Figure S11.** Spectral data of VLP H, **5**. **Figure S12.** Phenotypes of the *S. somaliensis* SCSIO ZH66 strains.

## Abbreviations

*wbl*: *whiB*-like
VLP: violapyrone
MRSA: methicillin-resistant *Staphylococcus aureus*
PKS: polyketide synthase
PAPS: 3′-phosphoadenosine 5′-phosphosulfate
ACP: acyl carrier protein
HPLC: high-pressure liquid chromatography
HR-ESI–MS: high-resolution electrospray mass spectrometry
THNS: 1,3,6,8-tetrahydroxynaphthaene synthase
MIC: minimum inhibitory concentration
qPCR: quantitative real-time RT-PCR
EMSA: electrophoretic mobility shift assay
MS: mannitol-soy flour
LB: Luria–Bertani
TSB: tryptic soy broth
