# Supplementary material for: Activation of a plasmid-situated type III PKS gene cluster by deletion of a wbl gene in deepsea-derived Streptomyces somaliensis SCSIO ZH66
Source: Microb Cell Fact. 2016 Jun 27;15:116. doi: 10.1186/s12934-016-0515-6 (PMC4924298; doi:10.1186/s12934-016-0515-6)
Supplement: Supplementary file 1 — 10.1186/s12934-016-0515-6 Anti-MRSA activities of violapyrones (VLPs 1–5). Table S2. Bacteria and plasmids used in this study. Table S3. The primer pairs used for cosmid library screening. Table S4. The primer pairs used for PCR-targeted mutagenesis. Table S5. The primer pairs used for PCR confirmation of the mutants. Table S6. The primer pairs used for qPCR analysis. Figure S1. Inactivation of wblA so. Figure S2. Spectral data of VLP B, 1. Figure S3. Inactivation of pksIII-1. Figure S4. Inactivation of vioA. Figure S5. Inactivation of vioB. Figure S6. Inactivation of orf1. Figure S7. Inactivation of orf(-1-2). Figure S8. Spectral data of VLP J, 3. Figure S9. Spectral data of VLP A, 2. Figure S10. Spectral data of VLP C, 4. Figure S11. Spectral data of VLP H, 5. Figure S12. Phenotypes of the S. somaliensis SCSIO ZH66 strains. [file 12934_2016_515_MOESM1_ESM.doc]

Supporting Information

**Activation of a plasmid-situated type III PKS gene cluster by deletion of a global regulatory *wbl* gene in deepsea-derived *Streptomyces somaliensis* SCSIO ZH66**

Huiming Huang^1^, Lukuan Hou^1^, Huayue Li^1^, Yanhong Qiu^1^, Jianhua Ju^2^ and Wenli Li^1^*

^1^ Key Laboratory of Marine Drugs, Ministry of Education of China, School of Medicine and Pharmacy, Ocean University of China, Qingdao 266003, China

^2^ CAS Key Laboratory of Marine Bio-resources Sustainable Utilization, Guangdong Key Laboratory of Marine Materia Medica, RNAM Center for Marine Microbiology, South China Sea Institute of Oceanology, Chinese Academy of Sciences, 164 West Xingang Road, Guangzhou 510301, China

***** Author to whom correspondence should be addressed

Email addresses:

HH: hmhuang1988@163.com

LH: 727052874@qq.com

HL: lihuayue@ouc.edu.cn

YQ: qq2yanhong@163.com

JJ: jju@scsio.ac.cn

WL: liwenli@ouc.edu.cn

Table of contents

| No. |  | Page |
| --- | --- | --- |
| 1． | **Table S1.** Anti-MRSA activities of violapyrones (VLPs **1**-**5**). | S3 |
| 2. | **Table S2.** Bacteria and plasmids used in this study. | S3 |
| 3. | **Table S3.** The primer pairs used for cosmid library screening. | S3 |
| 4. | **Table S4.** The primer pairs used for PCR-targeted mutagenesis. | S4 |
| 5. | **Table S5.** The primer pairs used for PCR confirmation of the mutants. | S4 |
| 6. | **Table S6.** The primer pairs used for qPCR analysis. | S4 |
| 7. | **Figure S1.** Inactivation of *wblA_so_*. | S5 |
| 8. | **Figure S2.** Spectral data of VLP B, **1**. | S5 |
| 9. | **Figure S3**. Inactivation of *pksIII-1*. | S7 |
| 10. | **Figure S4.** Inactivation of *vioA*. | S7 |
| 11. | **Figure S5.** Inactivation of *vioB*. | S8 |
| 12. | **Figure S6.** Inactivation of *orf1*. | S8 |
| 13. | **Figure S7.** Inactivation of *orf(-1-2)*. | S9 |
| 14. | **Figure S8.** Spectral data of VLP J, **3**. | S9 |
| 15. | **Figure S9.** Spectral data of VLP A, **2**. | S12 |
| 16. | **Figure S10.** Spectral data of VLP C, **4**. | S13 |
| 17. | **Figure S11.** Spectral data of VLP H, **5**. | S14 |
| 18. | **Figure S12.** Phenotypes of the *S. somaliensis* SCSIO ZH66 strains. | S15 |
| 19. | **References** | S15 |

**Table S1.** Anti-MRSA activities of violapyrones (VLPs **1**-**5**).

|  | Compounds | | | | | |
| --- | --- | --- | --- | --- | --- | --- |
|  | **1** | **2** | **3** | **4** | **5** | tetracycline |
| MIC (μg/mL) | 75 | >125 | 125 | 50 | 25 | 6.25 |

**Table S2.** Bacteria and plasmids used in this study.

| Strains or plasmids | Description | Reference or source |
| --- | --- | --- |
| Strains |  |  |
| *E. coli* Top10 | Host strain of cosmid vector SuperCos1 | Invitrogen |
| *E. coli* DH5*a* | Host strain for general cloning | Stratagene |
| *E. coli* ET12567/pUZ8002 | Host strain for conjugation | [1] |
| *E. coli* BW25113/pIJ790 | Host strain for PCR-targeting | [2] |
| *S. somaliensis* SCSIO ZH66 | Wild type, isolated from deepsea sendiment | [3] |
| *ΔwblA_so_* | *wblA_so_* inactivation mutant of *S. somaliensis* SCSIO ZH66 | This study |
| *ΔpksIII-1* | *pksIII-1* inactivation mutant of *S. somaliensis* SCSIO ZH66 | This study |
| *ΔvioA* | *vioA* inactivation mutant of *S. somaliensis* SCSIO ZH66 | This study |
| *ΔvioB* | *vioB* inactivation mutant of *S. somaliensis* SCSIO ZH66 | This study |
| *Δorf1* | *orf1* inactivation mutant of *S. somaliensis* SCSIO ZH66 | This study |
| *Δorf(-1-2)* | *orf(-1-2)* inactivation mutant of *S. somaliensis* SCSIO ZH66 | This study |
| Plasmids |  |  |
| SuperCosI | Ap^r^, Km^r^ , cosmid vector | Stratagene |
| pIJ773 | Apr^r^, source of *acc(3)IV*-*oriT* cassette | [4] |
| pIJ790 | Cm^r^, λ RED recombination plasmid | [4] |
| pWLI701 | cosmid harboring *wblA_so_* gene from *S. somaliensis* SCSIO ZH66 | This study |
| pWLI702 | pWLI701 derivative where *wblA_so_* was replaced with *acc(3)IV*-*oriT* cassette | This study |
| pWLI711 | cosmid harboring *pksIII-1* gene from *S. somaliensis* SCSIO ZH66 | This study |
| pWLI712 | pWLI711 derivative where *pksIII-1* was replaced with *acc(3)IV*-*oriT* cassette | This study |
| pWLI801 | cosmid harboring *vio* genes from *S. somaliensis* SCSIO ZH66 | This study |
| pWLI802 | pWLI801 derivative where *vioA* was replaced with *acc(3)IV*-*oriT* cassette | This study |
| pWLI803 | pWLI801 derivative where *vioB* was replaced with *acc(3)IV*-*oriT* cassette | This study |
| pWLI804 | pWLI801 derivative where *orf1* was replaced with *acc(3)IV*-*oriT* cassette | This study |
| pWLI805 | pWLI801 derivative where *orf(-1-2)* was replaced with *acc(3)IV*-*oriT* cassette | This study |

**Table S3.** The primer pairs used for cosmid library screening.

| cosmid | Primer pairs used for cosmid library screening (5'-3') |
| --- | --- |
| pWLI701 | *wblA_so_*SF:GAAGGTCCCGCATGTCCTCA  *wblA_so_*SR:TGCGGAGCTGTCGTTCACCT |
| pWLI711 | *pksIII-1*SF:atgttgccccgttcggtga  *pksIII-1*SR:aacgtcctgatcgtggcct |
| pWLI801 | *vioA*SF:ccgagatgatccgctgact  *vioA*SR:acatagccgagcaagctgcc |

**Table S4.** The primer pairs used for PCR-targeted mutagenesis^a^.

| gene | Primer pairs used for inactivation (5'-3') |
| --- | --- |
| *wblA_so_* | *wblA_so_*MF:tatgggctgggtgaccgactggagtgcgcaggccgcctgcattccggggatccgtcgacc  *wblA_so_*MR:gtctagccgacggcgagcagccgcgcgtagacctcgcggtctgtaggctggagctgcttc |
| *vioA* | *vioA*MF: CATGGCCATCCACATCGCCCAGCCCACCACCATCCTCGGCattccggggatccgtcgacc  *vioA*MR: TGTCACGCCGCCCAGACCCCACGGATCGCTGCCGTGTTGAAtgtaggctggagctgcttc |
| *pksIII-1* | *pksIII-1*MF:CATGGCCGTTCTCTGCCGTCCCGCCGTTGCCGTCCCGCCGattccggggatccgtcgacc *pksIII-1*MR:GCTCAGGCCGGGCGGCGTGCCTCGGCGCCGGTCAGGGCGGCtgtaggctggagctgcttc |
| *vioB* | *vioB*MF: CATGCACAAAACCGCCCTCCAAGCGCTCCTCCAAGAACGCattccggggatccgtcgacc  *vioB*MR: GGCTAGCGGGCTCGGAGCATCGGTGTGCGGGCGTGCGCACGtgtaggctggagctgcttc |
| *orf1* | *orf1*MF: CATGACCGCCATCCCTCTCGTGCAGGGCCGCTGCCCCGCCattccggggatccgtcgacc  *orf1*MR: GGTCAGGCGGCGAGGTAGCCGGGGCAGTGGACGGGCTCCATtgtaggctggagctgcttc |
| *orf(-1-2)* | *orf(-1-2)*MF: GGACACCGTGTCGACTCTTCTGTTCCCGCTCCGAGCCCAGattccggggatccgtcgacc  *orf(-1-2)*MR:CAGCAGCCGGGCGTAGGCTGCCGGGCACCACAAGCAGCAGGtgtaggctggagctgcttc |

^a^Underlined letters represent nucleotides homologous to the DNA regions internal to target genes

**Table S5.** The primer pairs used for PCR confirmation of the mutants.

| gene | Primer pairs designed to verify the mutant strains (5'-3') | Fragment  Replaced | Length of desired PCR fragments | |
| --- | --- | --- | --- | --- |
|  |  |  | Wild type | Mutant |
| *wblA_so_* | *wblA_so_*CF: GTATCCACAACTGTCACAGCACCG  *wblA_so_*CR: TTCCGGTTCCGGTCGAGGAGTT | 375 bp | 1214 bp | 2221 bp |
| *vioA* | *vioA*CF: CAGGCGAGGTCGACCACA  *vioA*CR: AGCCCAGGCAACGATCAA | 1053 bp | 1251 bp | 1580 bp |
| *pksIII-1* | *pksIII-1*CF: GCGTGGAGGGCAGCAGGT  *pksIII-1*CR: CCCAAGACGCGTTCGCGC | 1098 bp | 1237 bp | 1521 bp |
| *vioB* | *vioB*CF: GCGATGTGGATGGCCATGGT  *vioB*CR: TGTCACGAGACGGCCACAAC | 819 bp | 1176 bp | 1739 bp |
| *orf1* | *orf1*CF: GGGAAGCCGTGTGGGCGGTC  *orf1*CR: GACCGCATCGCCGCCATCCT | 477 bp | 573 bp | 1478 bp |
| *orf(-1-2)* | *orf(-1-2)*CF: CAACGTTCTCGACATCAGTC  *orf(-1-2)*CR: GTGCTGCGGATTTTGGAG | 451 bp | 640 bp | 1571 bp |

**Table S6.** The primer pairs used for qPCR analysis.

| gene | Primer pairs used for inactivation (5'-3') |
| --- | --- |
| *vioA* | FP: GACAGGGCTTCCTTCGTGGA  RP: GATCTACAAGGCGCTGTTCGG |
| *vioB* | FP: ATGCGGTGGATGCTGTGG  RP: CGTCGAGCCCTGCGTGTAT |
| *whiA* | FP: TGGTGAGCGGCCGCATCG  RP: GTCGACCAGCCCCGTCTG |
| *whiB* | FP: ACAACTGCTGGTCGAGGAA  RP: CCGAACGGACTTCACAGG |
| *whiD* | FP: AGCTCCCTCTTCTTCCACCC  RP: GCTCTTCGCGTTCGTCCTC |
| *whiG* | FP: CGGGCGATCAACCTCCT  RP: GCAGAACCGATTTCGTATGG |
| *whiH* | FP: CGAACTCGACCGCTACCCC  RP: CGGCCGCGATTACTGCTC |
| *whiI* | FP: CCTACCGCCCGAACAAGC  RP: CCTCCGCAATGGACGATG |
| *wblC* | FP: CGGAGGATTCCACCTTGTCT  RP: GCAGAGGGACTTGGCGTATT |
| *wblE* | FP: TCTTCTTCCCCATCGGCAAC  RP: GCTCGTCCTCGCTGAGGC |
| *wblH* | FP: GCGGATCGACAGTACGGC  RP: CCTCGCAGATGAGCTTGGC |
| *wblI* | FP: CGGGTCTGTGCCCAGTGTC  RP: TATCGGGCCGGAGGTGA |
| *wblK* | FP: ATCGTGGCCCGCTTCCT  RP: CGTCGTATCCAGCCGCTCA |
| *hrdB* | FP: CGACTACACCAAGGGCTACAA  RP: GGAGCATCTGACGCTGGAC |

**A)**

**B)**


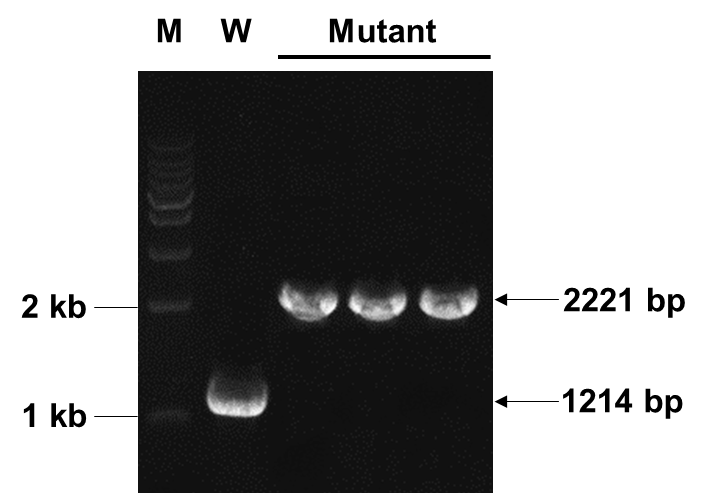


**Figure S1.** Inactivation of *wblA_so_*. (A) Construction of *wblA_so_* gene inactivation mutant. (B) PCR confirmation of the double-crossover mutant. M: DNA marker; W: *S. somaliensis* SCSIO ZH66 wild type strain; Mutant: *wblA_so_* gene inactivation mutant.

**Figure S2.** Spectral data of VLP B, **1**.


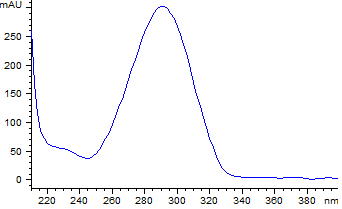


**Figure S2 (A)**. UV spectrum of **1.**

**
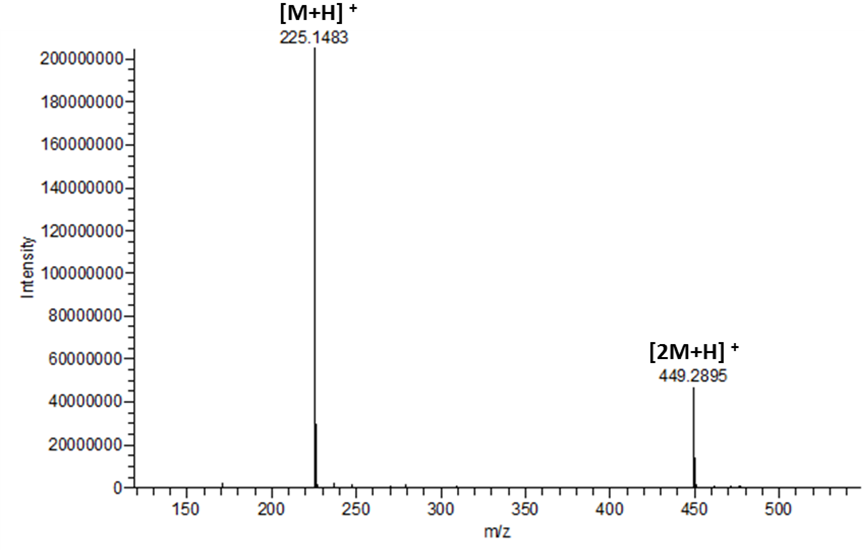
**

**Figure S2 (B)**. The HRMS spectrum of **1.**


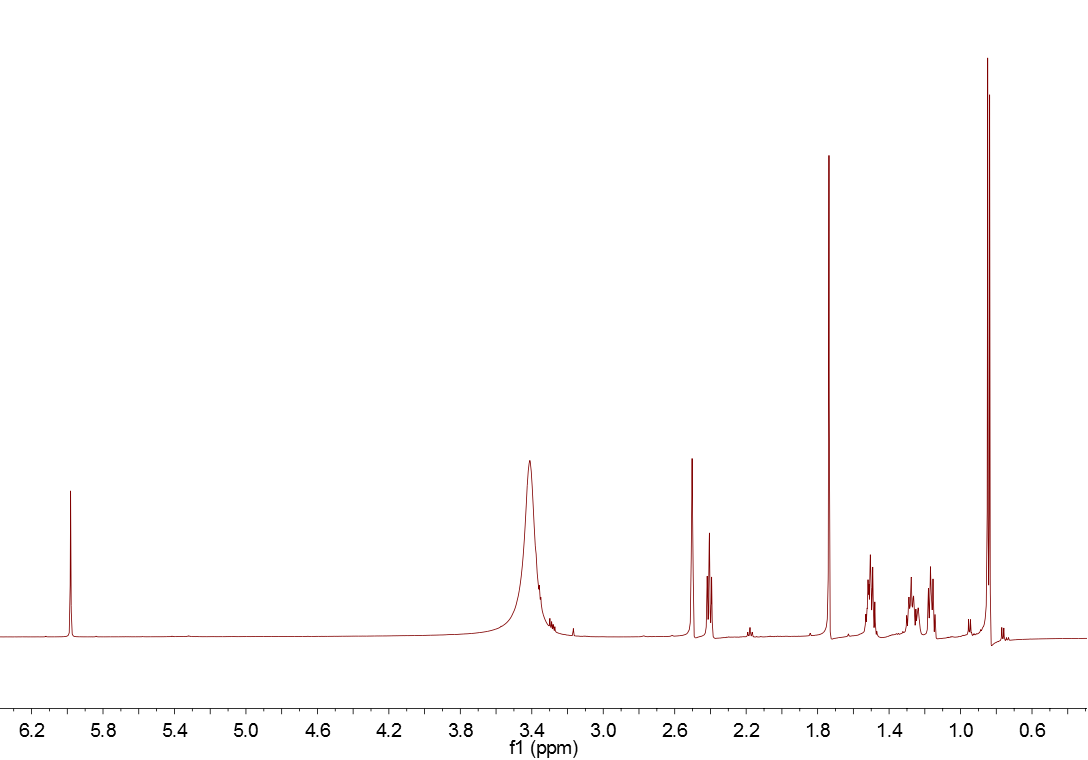


**Figure S2 (C)**. The ^1^H NMR spectrum of **1** in DMSO-*d_6_*.

**A)**

**B)**


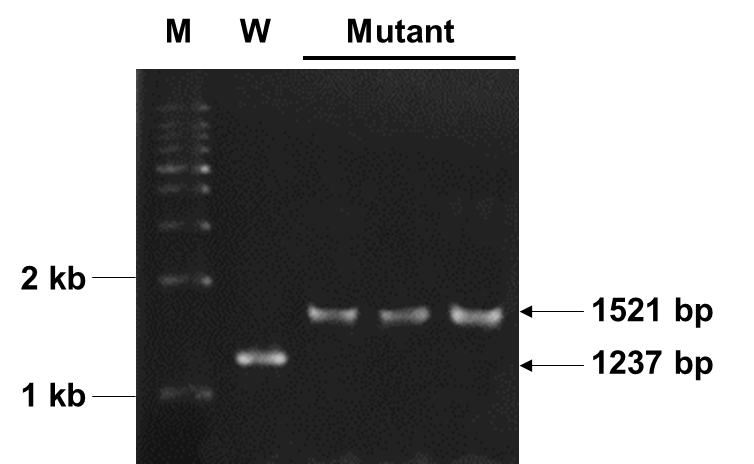


**Figure S3.** Inactivation of *pksIII-1*. (A) Construction of *pksIII-1* gene inactivation mutant. (B) PCR confirmation of the double-crossover mutant. M: DNA marker; W: *S. somaliensis* SCSIO ZH66 wild type strain; Mutant: *pksIII-1* gene inactivation mutant.

**A)**

**B)**


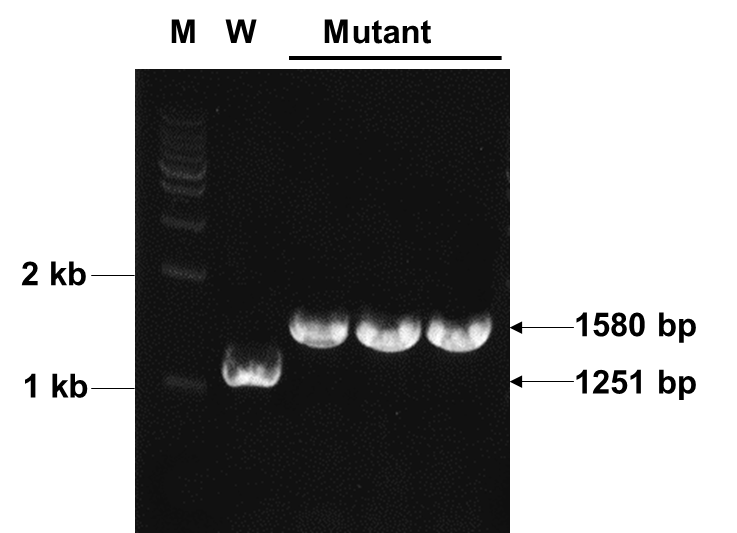


**Figure S4.** Inactivation of *pksIII-2* (*vioA*). (A) Construction of *pksIII-2* (*vioA*) gene inactivation mutant. (B) PCR confirmation of the double-crossover mutant. M: DNA marker; W: *S. somaliensis* SCSIO ZH66 wild type strain; Mutant: *pksIII-2* (*vioA*) gene inactivation mutant.

**A)**

**B)**


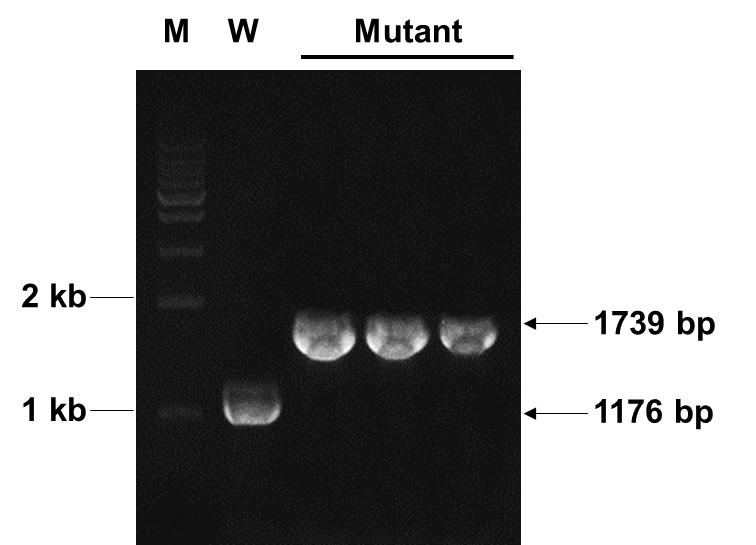


**Figure S5.** Inactivation of *vioB*. (A) Construction of *vioB* gene inactivation mutant. (B) PCR confirmation of the double-crossover mutant. M: DNA marker; W: *S. somaliensis* SCSIO ZH66 wild type strain; Mutant: *vioB* gene inactivation mutant.

**A)**

**B)**


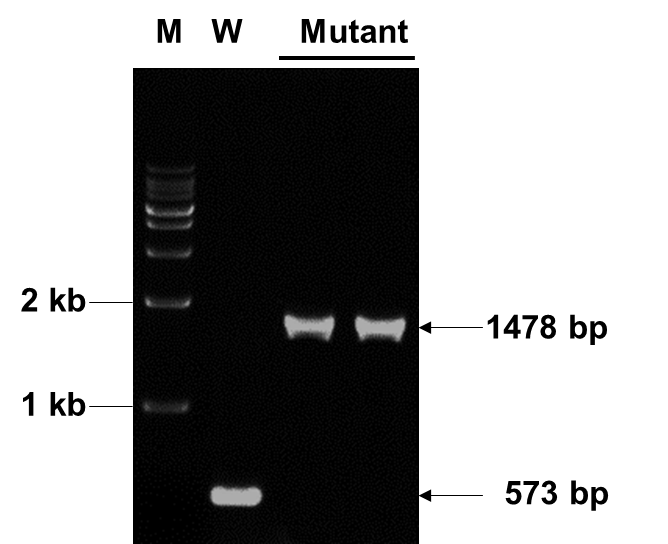


**Figure S6.** Inactivation of *orf1*. (A) Construction of *orf1* gene inactivation mutant. (B) PCR confirmation of the double-crossover mutant. M: DNA marker; W: *S. somaliensis* SCSIO ZH66 wild type strain; Mutant: *orf1* gene inactivation mutant.

**A)**

**B)**


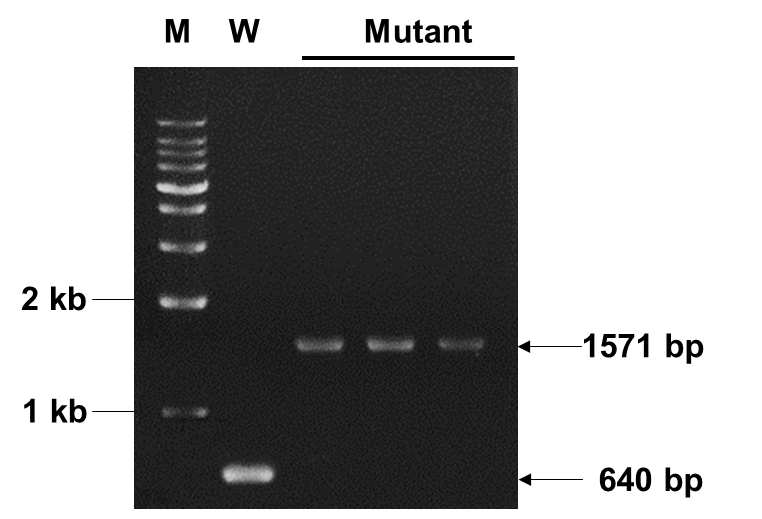


**Figure S7.** Inactivation of *orf(-1-2)*. (A) Construction of *orf(-1-2)* gene inactivation mutant. (B) PCR confirmation of the double-crossover mutant. M: DNA marker; W: *S. somaliensis* SCSIO ZH66 wild type strain; Mutant: *orf(-1-2)* gene inactivation mutant.

**Figure S8.** Spectral data of VLP J, **3**.

**
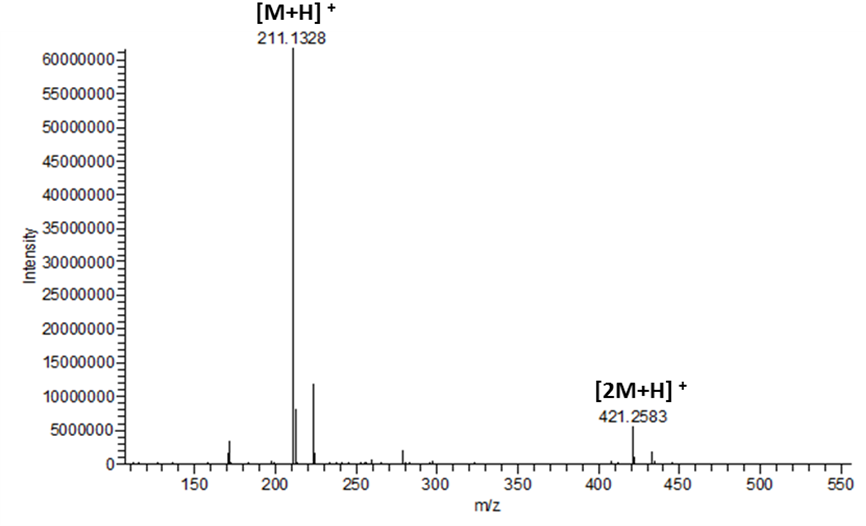
**

**Figure S8.** **(A)** The HRMS spectrum of **3.**


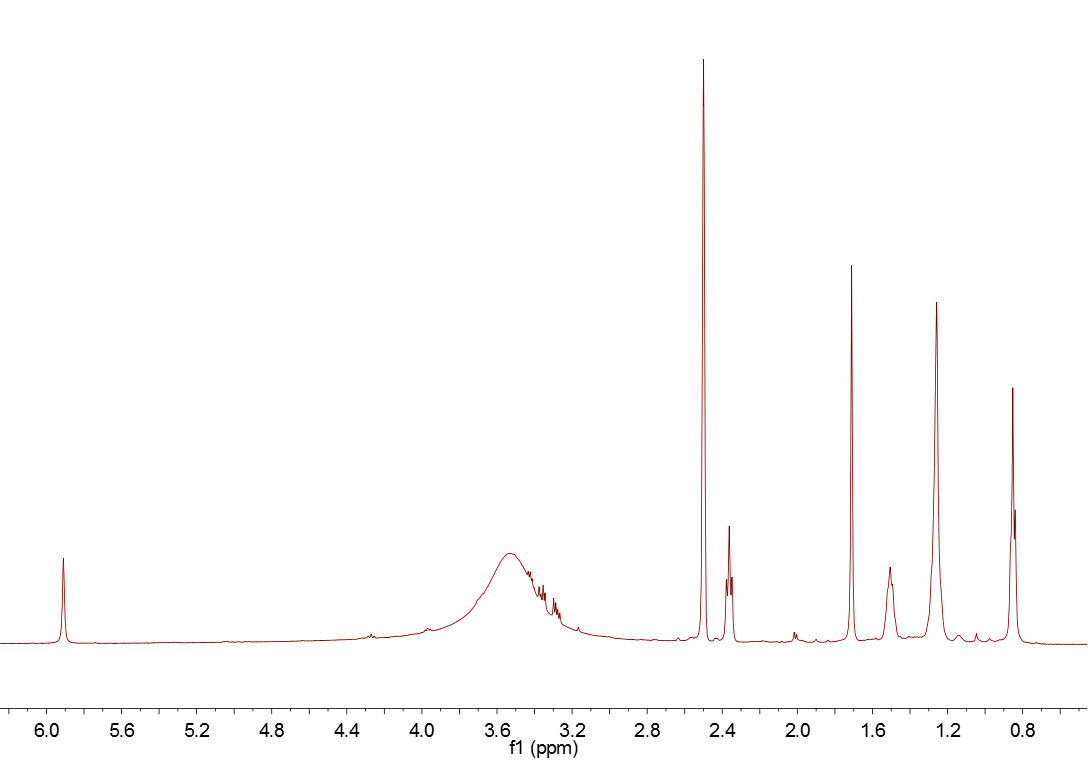


**Figure S8. (B)** The ^1^H NMR spectrum of **3** in DMSO-*d_6_*.


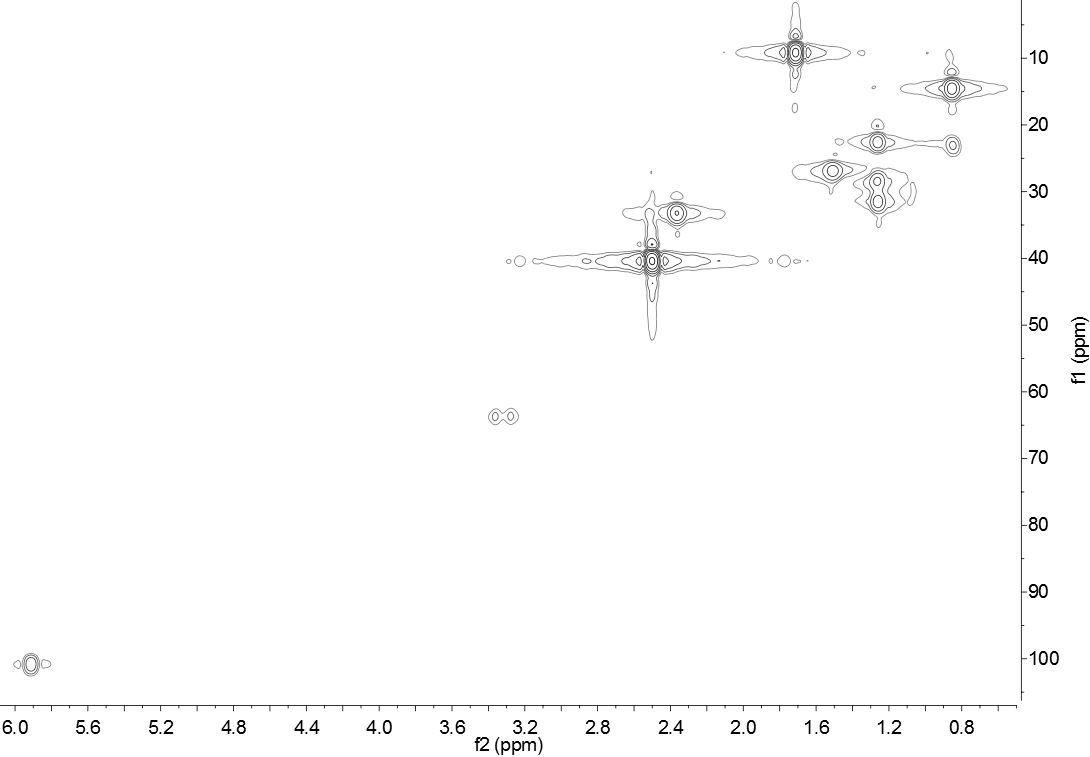


**Figure S8. (C)** The HSQC spectrum of **3** in DMSO-*d_6_*.


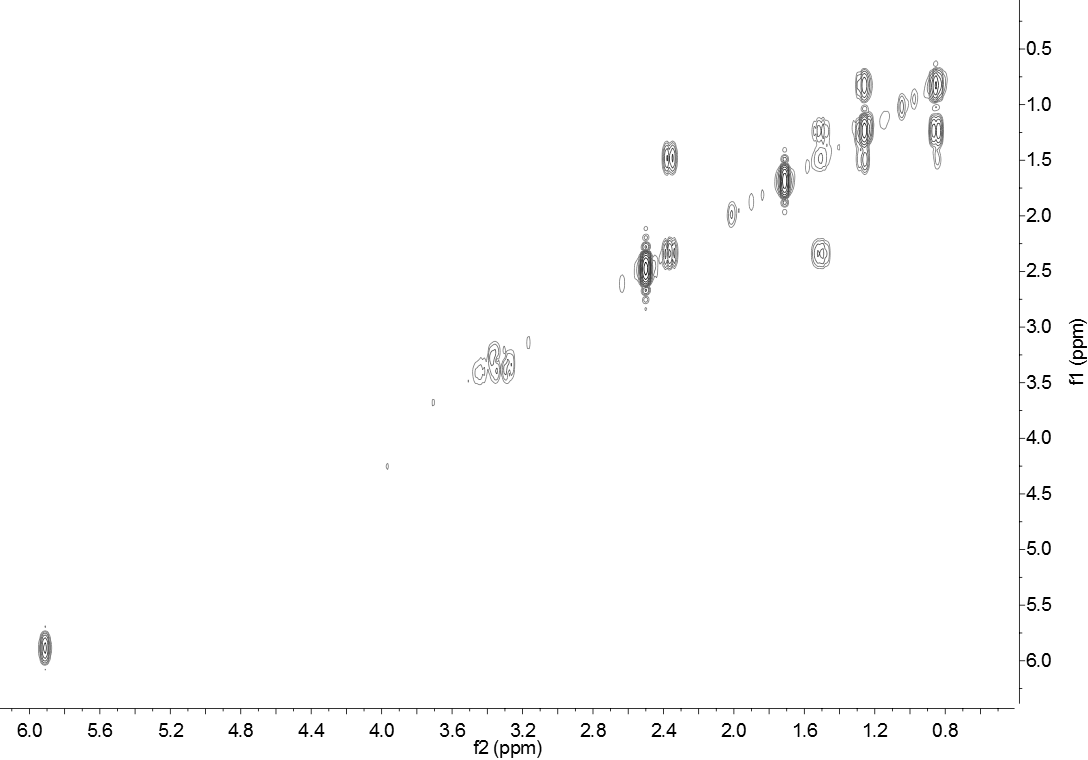


**Figure S8. (D)** The ^1^H-^1^H COSY spectrum of **3** in DMSO-*d_6_*.


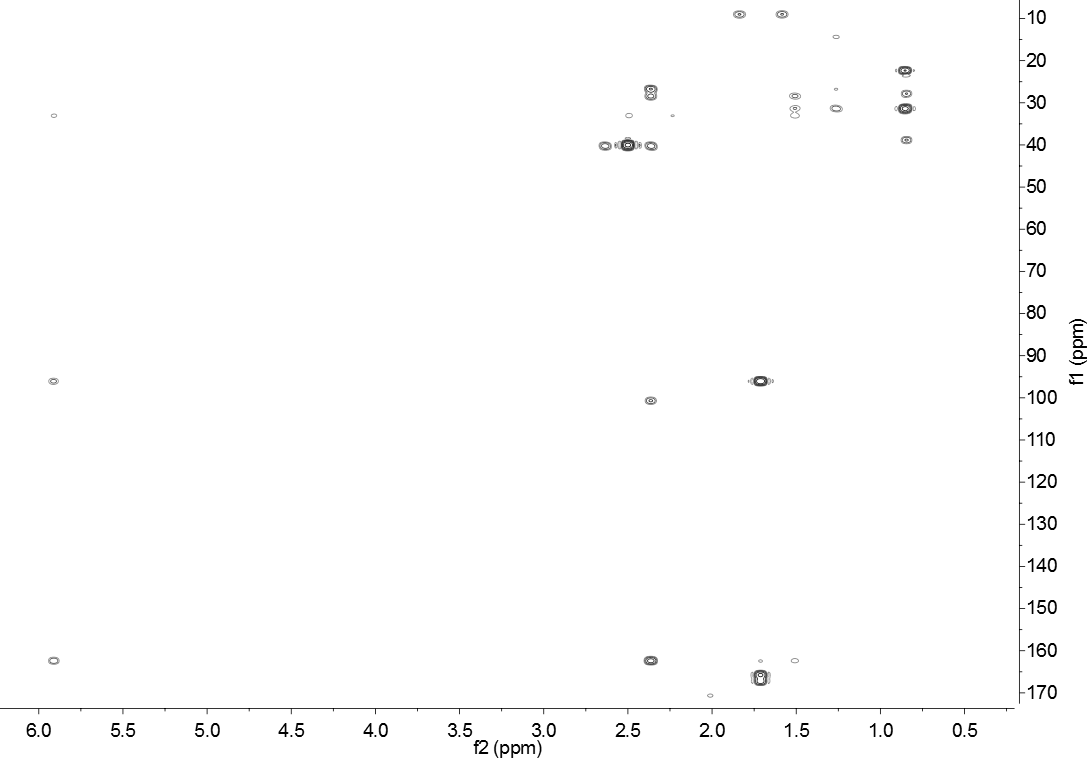


**Figure S8. (E)** The HMBC spectrum of **3** in DMSO-*d_6_*.

**Figure S9.** Spectral data of VLP A, **2**.

**
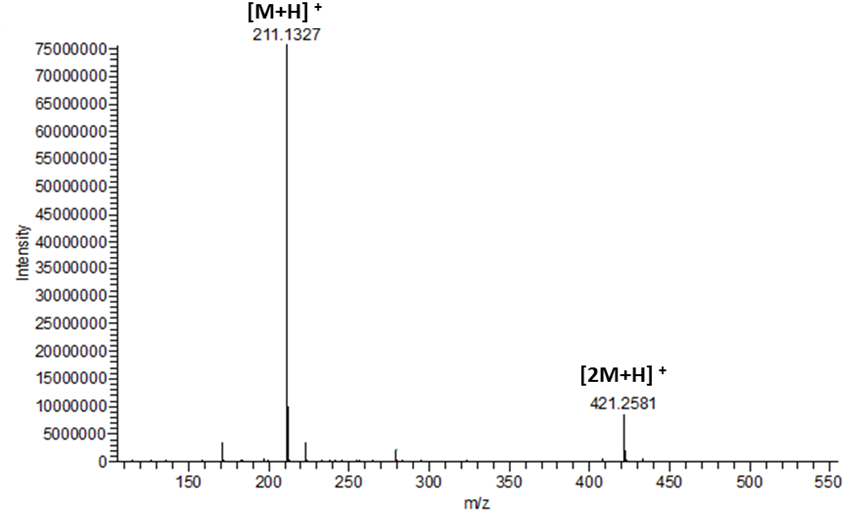
**

**Figure S9. (A)** The HRMS spectrum of **2.**


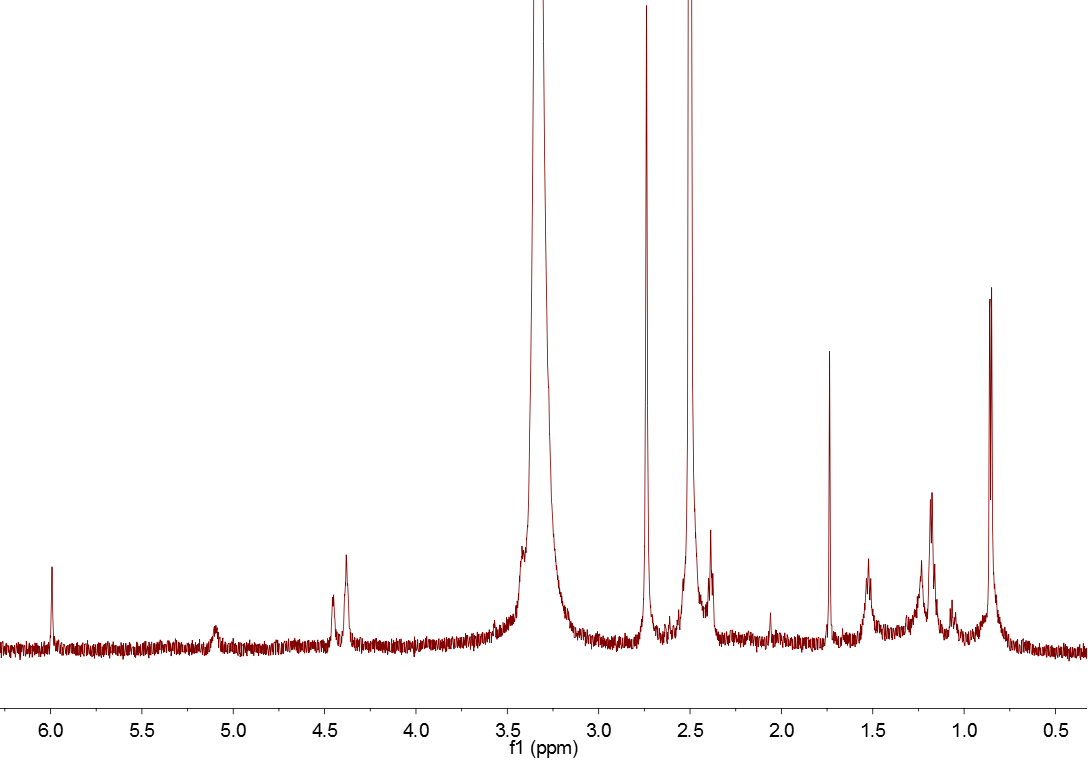


**Figure S9. (B)** The ^1^H NMR spectrum of **2** in DMSO-*d_6_*.

**Figure S10.** Spectral data of VLP C, **4**.

**
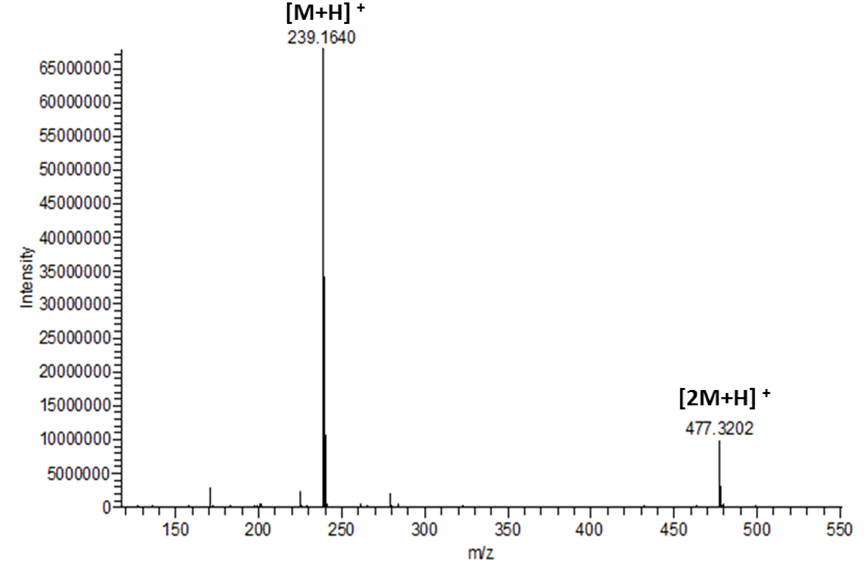
**

**Figure S10. (A)** The HRMS spectrum of **4**.


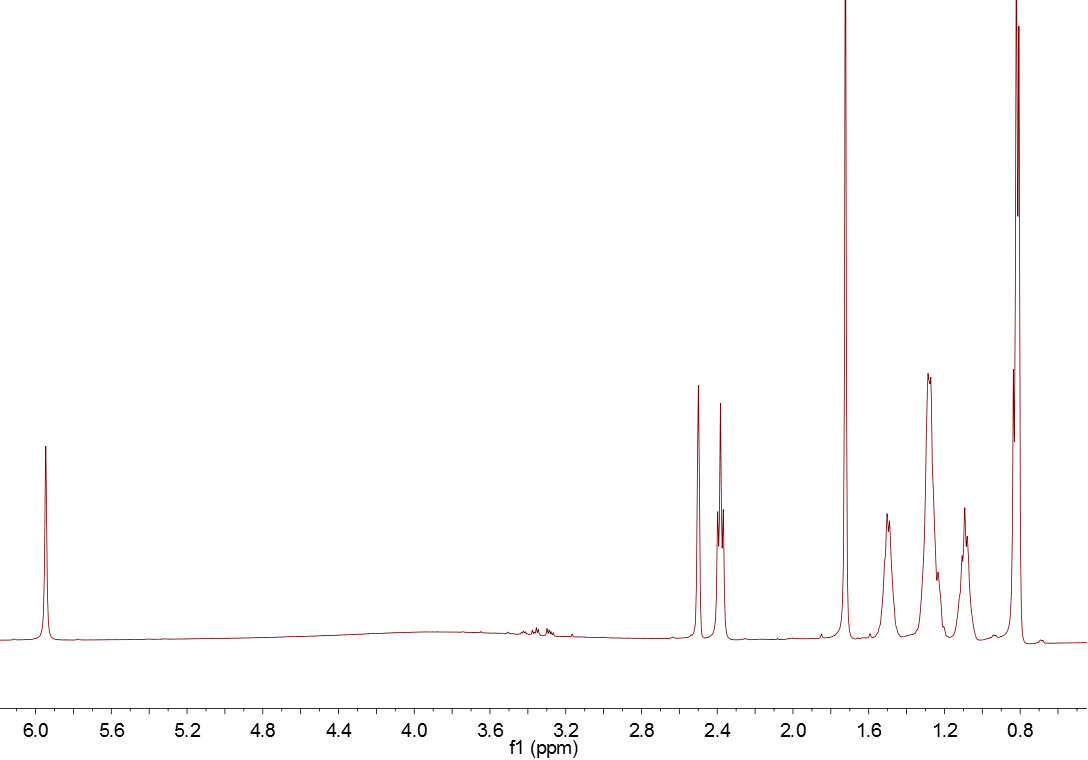


**Figure S10. (B)** The ^1^H NMR spectrum of **4** in DMSO-*d_6_*.

**Figure S11.** Spectral data of VLP H, **5**.

**
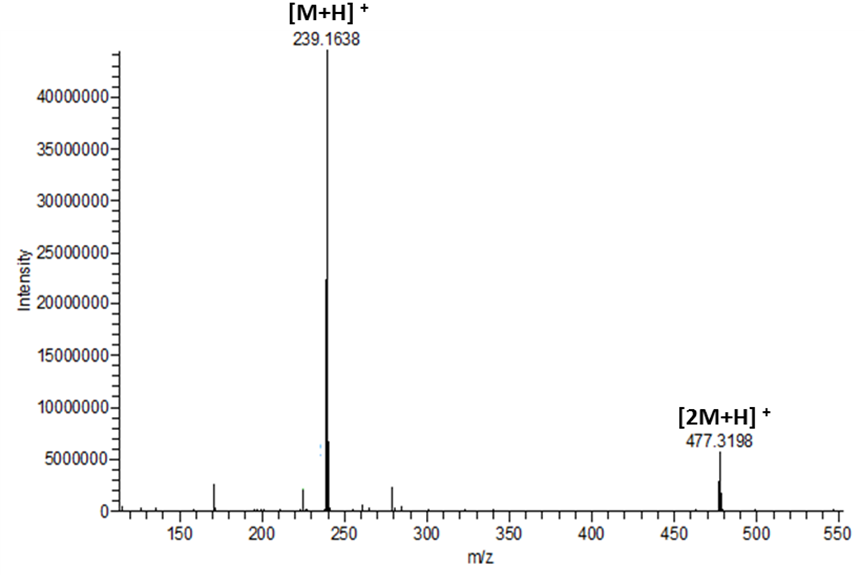
**

**Figure S11. (A)** The HRMS spectrum of **5**.


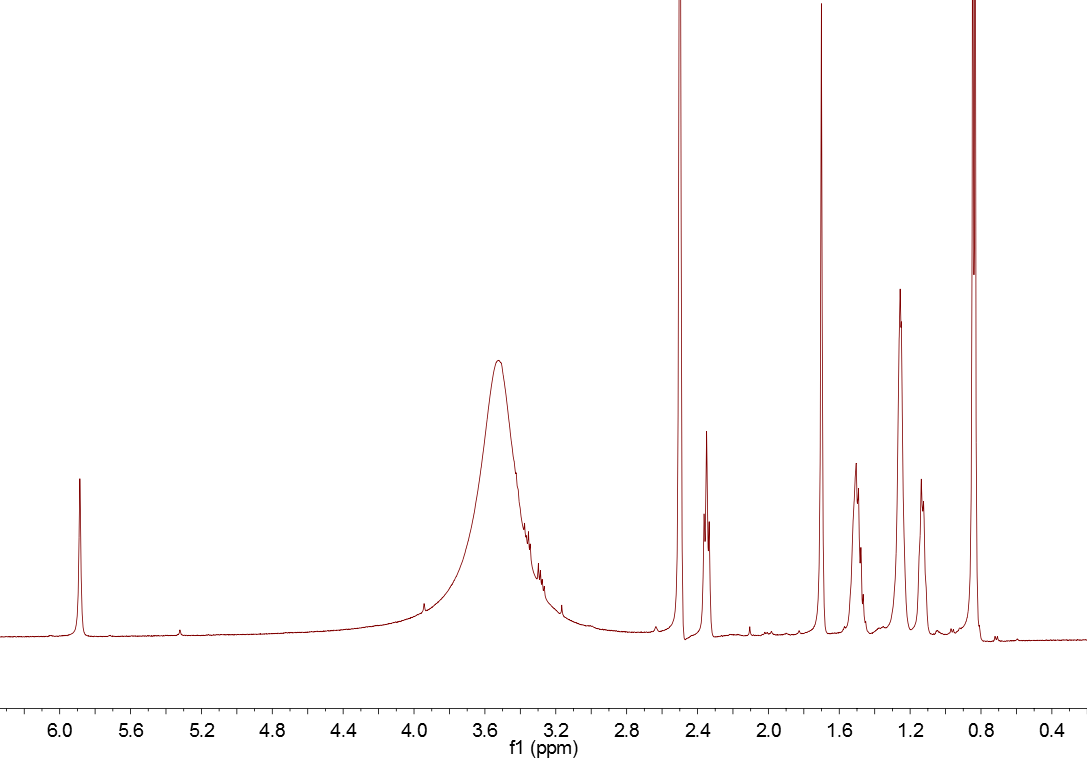


**Figure S11. (B)** The ^1^H NMR spectrum of **5** in DMSO-*d_6_*.


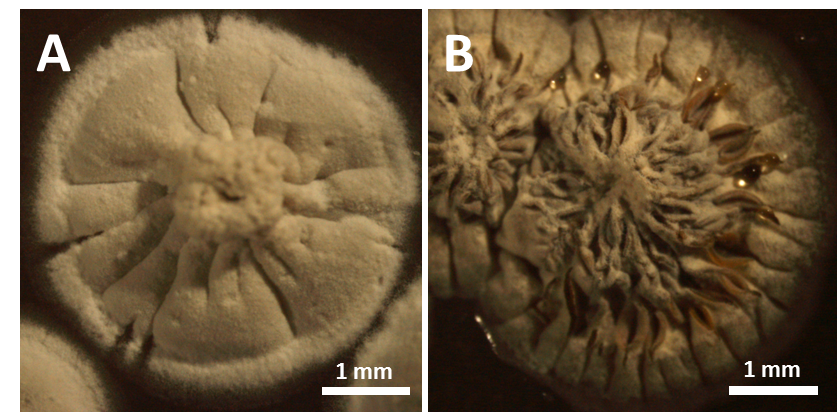


**Figure S12.** Phenotypes of the *S. somaliensis* SCSIO ZH66 strains. (A) the wild-type strain; (B) the *ΔwblA_so_* mutant. Images were recorded with a Nikon ECLIPSE 80i microscope equipped with a digital camera (Melville, NY). Strains were incubated on MS plates at 30 °C for 4 days. Bar, 1 mm.

**References**

1. Datsenko KA, Wanner BL. One-step inactivation of chromosomal genes in *Escherichia coli* K-12 using PCR products. Proc Natl Acad Sci USA. 2000;97(12):6640-5.

2. MacNeil DJ, Gewain KM, Ruby CL, Dezeny G, Gibbons PH, MacNeil T. Analysis of *Streptomyces avermitilis* genes required for avermectin biosynthesis utilizing a novel integration vector. Gene. 1992;111(1):61-8.

3. Zhang Y, Huang H, Xu S, Wang B, Ju J, Tan H, Li W. Activation and enhancement of Fredericamycin A production in deepsea-derived *Streptomyces somaliensis* SCSIO ZH66 by using ribosome engineering and response surface methodology. Microb Cell Fact. 2015;14:64.

4. Gust B, Challis GL, Fowler K, Kieser T, Chater KF. PCR-targeted *Streptomyces* gene replacement identifies a protein domain needed for biosynthesis of the sesquiterpene soil odor geosmin. Proc Natl Acad Sci USA. 2003;100(4):1541-6.
